# The Baby Care Questionnaire: A measure of parenting principles and practices during infancy^☆^

**DOI:** 10.1016/j.infbeh.2013.08.004

**Published:** 2013-12

**Authors:** Alice Winstanley, Merideth Gattis

**Affiliations:** School of Psychology, Cardiff University, Cardiff, United Kingdom

**Keywords:** Parenting, Infancy, Attunement, Structure, Infant crying, Breastfeeding, Bed-sharing

## Abstract

The current report provides a new framework to explore the role of parenting practices and principles during infancy. We identify structure and attunement as key parenting principles during infancy. Structure represents reliance on regularity and routines in daily life. Attunement represents reliance on infant cues and close physical contact. We suggest parents’ relative endorsement of these parenting principles is related to their choices about practices such as feeding, holding and night-time sleeping. We designed the Baby Care Questionnaire to measure parents’ endorsement of structure and attunement, as well as their daily parenting practices. We report data demonstrating the factor structure, reliability and validity of the BCQ. The BCQ, to our knowledge, is the first comprehensive measure of parenting practices and principles during infancy. We conclude with a discussion of future directions for the measure.

## Highlights

• The Baby Care Questionnaire (BCQ) measures parents’ support of parenting principles.
• The BCQ measures two factors (principles) – structure and attunement.
• The BCQ showed reliability – internal consistency and test–retest reliability.
• Structure and attunement were related to parenting practices, such as breastfeeding.
• Structure and attunement were related to perceived control over caregiving failures.

## 1 Introduction

Every day, caregivers around the world make decisions about how to care for their infants. Caregiving decisions differ within and across families and cultures, despite all babies’ biological similarity (Small, 1999). We propose that caregiving decisions are based on general principles, and are reflected in practices or specific behaviours parents use to achieve positive outcomes for their offspring. In the current report, we address the need for empirical investigations of caregiving principles and practices in infancy and introduce a new measure, the Baby Care Questionnaire, to be used in such investigations.

### 1.1 Caregiving during infancy

Infancy is a period of high dependency and intense caregiving in which parents must respond to their infant's need for food, sleep and emotional attachment (Bornstein, 2002; Small, 1999). Parents differ in their beliefs about the best way to meet these needs as well as their specific caregiving behaviours. Research on caregiving principles and practices has tended to focus on specific infant needs, such as eating, sleeping, or soothing, rather than looking across those needs. For example, the Infant Feeding Style Questionnaire (IFSQ; Thompson et al., 2009) and the Caregiver's Feeding Style Questionnaire (CFSQ; Hughes et al., 2012; Hughes, Power, Orlet Fisher, Mueller, & Nicklas, 2005) are parent-report measures of parental beliefs and behaviours related to feeding, and have been used to identify specific strategies for feeding as well as their correlates and developmental consequences. Similarly, the Brief Infant Sleep Questionnaire (Sadeh, 2004) and the Parental Interactive Bedtime Behaviour Scale (Morrell & Cortina-Borja, 2002) are parent-report measures of parent and infant behaviours surrounding bedtime and sleeping, and have been used to identify relations between parent and infant behaviours, such as parenting bedtime routines and infant sleeping problems. A small number of studies have examined parenting practices across different domains. However, such studies have focused on specific associations between practices, such as the association between bed-sharing and breastfeeding (Ball, Hooker, & Kelly, 1999; Blair, Heron, & Fleming, 2010; Santos, Mota, Matijasevich, Barros, & Barros, 2009), rather than on general caregiving principles and potential relations between principles and caregiving practices during infancy.

We propose that two key principles that guide caregiving during infancy are structure and attunement. *Structure* refers to the extent to which parents endorse the utilisation of regularity and routines in infant care. Many popular books and magazines aimed at parents have argued for the benefits of regularity and routines in caring for infants (for example, Ford, 2001), but to date few empirical studies have investigated the influence of parental support for regularity and routines during infancy on developmental outcomes. A number of studies have investigated the influence of regularity and routines during childhood on developmental outcomes and reported positive outcomes for both children and parents (see reviews from Boyce, Jensen, James, & Peacock, 1983; Fiese et al., 2002; Grusec & Davidov, 2010; Spagnola & Fiese, 2007). For example, routines in daily life are associated with better sleep for children and their parents (Mindell, Telofski, Wiegand, & Kurtz, 2009; Seymour, Brock, During, & Poole, 1989), elevated mood (Sprunger, Boyce, & Gaines, 1985), lower levels of conflict and greater life satisfaction (Jensen, James, Boyce, & Hartnett, 1983). In the context of parent-child interactions, the term structured parenting has been used to refer to varying degrees of parental control, direction and guidance, such as instructing a child in how to play a game, and it too is associated with positive developmental outcomes (Leve et al., 2009). In the context of infant care, the principle of structure also implies some degree of parental control and guidance, as infants are not able to autonomously regulate states such as sleeping and arousal. St James-Roberts (2007) has argued that sleeping schedules, a form of parental control, function as learning scaffolds for infants to learn sleeping and waking patterns and therefore can reduce night waking. Thus structure is a principle about the role of external guidance in helping infants increase the regularity of states such as sleeping and waking, hunger and eating, and arousal and soothing.

*Attunement* refers to the extent to which parents endorse the utilisation of infant cues and close physical contact in infant care. In the context of parent-child interactions, the term attunement has been used to describe parental sensitivity and responsiveness to infant cues and attentional states, such as noticing an infant's interest in an object, and describing the object (Bornstein, Tamis-LeMonda, Hahn, & Haynes, 2008; Legerstee, Markova, & Fisher, 2007). In the context of caregiving, attunement describes variations between caregivers in the extent to which they value and rely on children's cues to hunger and satiety, drowsiness and wakefulness, and distress and soothing (Hughes et al., 2012; Tikotzky & Sadeh, 2009). Previous studies have established relations between sensitivity and responsiveness to children's attentional states and interests and a number of positive developmental outcomes, such as social, communicative and cognitive skills (e.g., Bornstein & Tamis-LeMonda, 1997; Landry, Smith, & Swank, 2006; Legerstee et al., 2007). Few studies, however, have investigated relations between attunement in the context of caregiving and developmental outcomes: those that have done so have for the most part focused specifically on maternal responsivity to infant distress and its effect on infant crying (for example, Bell & Ainsworth, 1972; Hubbard & van Ijzendoorn, 1991). In this paper we aim to investigate the relations between attunement and caregiving practices more generally.

Individual differences in parental beliefs about infants and parenting have important effects on choice and effectiveness of parenting behaviours. For example, parents who assign more responsibility to the child than the adult for caregiving failures are more unwavering and directive in their parenting behaviour (Bugental & Johnston, 2000; Guzell & Vernon-Feagans, 2004). Such parents may endorse structure, as this principle allows parents to have more control and regularity in the caregiving role. Individual differences in caregiving beliefs may be due to caregiving experience: expectant parents evaluate and develop caregiving principles during pregnancy, but the experiences of caring for an infant may alter or strengthen these principles. Finally, individual differences in caregiving principles may also be due to infant characteristics such as age, gender, health and temperament.

Structure and attunement are often considered opposing categories – for example, caregiving experts tend to advocate either infant-demand or scheduled parenting (St James-Roberts, 2007; St James-Roberts et al., 2006). However, the negative relationship between structure and attunement, as well as the approaches advocated, are based on personal experience and popular culture (for example, “huggers” and “schedulers”; James, 2008; Williams, 2010) rather than empirical evidence (St James-Roberts, 2007). Middle-class parents in historically interdependent societies, such as Costa Rica, appear to combine scheduled and infant-demand caregiving (Kagitcibasi, 2005; Keller, Borke, Yovsi, Lohaus, & Jensen, 2005). We propose that structure and attunement are orthogonal and so caregiving principles can vary independently.

### 1.2 Relations between principles and practices

One of the questions examined in the current study was about the relations between parenting principles and daily decisions about practices, such as where to put infants to sleep, what to feed them and how long to hold them (specific hypotheses are discussed below). Identifying relations between parenting principles and practices is needed to help scientists and practitioners understand the independent and joint influence of these principles and practices on infant development. Such insight may be particularly useful in understanding how parents reason about health-related recommendations (see Ball & Volpe, 2013, for a review of the importance of beliefs on bed-sharing behaviours in response to public health messages). For example, UK parents continue to bed-share despite advice that the safest place for babies to sleep is in a cot near the parents’ bed (NHS choices, 2012; UNICEF UK Baby Friendly Initiative, 2013). In one study, no first-time UK parents planned to bed-share, but three to four months after birth 70% of parents were sharing a bed with their infant either habitually (every night) or occasionally (at least once a week; Ball et al., 1999). Similarly, although breastfeeding is widely recommended, an NHS survey of infant feeding showed a rapid decline in breastfeeding over the first few weeks and months of life from an initial rate of 70% (Bolling, Grant, Hamlyn, & Thornton, 2007). In addition, examining the effects of principles on behaviour is a crucial validation procedure (Holden & Edwards, 1989). Holden and Edwards (1989) claimed that beliefs must reflect behaviour to have consequences on children.

We hypothesised that parents who endorse attunement would be more likely to choose bed-sharing and breastfeeding as parenting practices and show longer durations of holding. Holding should be related to attunement due to endorsement of close physical contact. Bed-sharing refers to times parents and their infant(s) sleep in the same bed, whereas co-sleeping refers to infants sleeping in close proximity to their parents (Ball & Volpe, 2013). Bed-sharing has great historical (Nelson, Schiefenhoevel, & Haimerl, 2000), cultural (Blair, 2010) and functional diversity (McKenna, 1996; Wailoo, Ball, Flemming, & Platt, 2004). When explaining their sleeping practices, new parents often brought up the ease of nocturnal breastfeeding when paired with bed-sharing (Ball et al., 1999). Night-time observations have also shown this “natural relationship”, which exists in a high proportion of the world's societies (McKenna, Mosko, & Richard, 1997; Wailoo et al., 2004). McKenna et al. (1997) observed mothers and their healthy, exclusively breastfed 3- to 4-month-old infants during the night, and reported that infants who routinely co-slept breastfed for three times longer in the night than infants who routinely slept apart. However, bed-sharing with and without breastfeeding appear to differ (Volpe, Ball, & McKenna, 2013). Night-time observations suggested non-breastfeeding mothers seemed to share a bed like they were sharing with an adult, whereas breastfeeding mothers tended to adopt a different position by placing the infant at breast level, facing the mother. McKenna et al. (1997) suggested bed-sharing may allow mothers to sense and attend to subtle sounds and movements infants make with increasing frequency and intensity when approaching breastfeeding episodes (McKenna, 1996; McKenna et al., 1997). When infants were sleeping in a separate room, mothers were only able to sense frank crying. Parents who bed-share thus appear to be ensuring they can sense and respond to even the most subtle of infants’ cues, reflecting attunement.

We hypothesised that parents who endorse structure would be less likely to bed-share and breastfeed, and instead would report independent sleeping and formula feeding. UK parents are said to value infants sleeping through the night, encouraging regular sleeping times and acclimatisation to sleeping alone (Mindell, Sadeh, Wiegand, How, & Goh, 2010; St James-Roberts et al., 2006). St James-Roberts et al. (2006) reported that at 12 weeks, parents in a more scheduled London sample only averaged one night of bed-sharing per week and only 37% of parents were breastfeeding. These figures are in line with the NHS survey on infant feeding, which reported breastfeeding rates dropping from 48% at 6 weeks to 25% by 6 months (Bolling et al., 2007). Therefore, parents in the UK – a culture where conventional values are consistent with the parenting principle of structure – appear less likely to bed-share and breastfeed.

### 1.3 The Baby Care Questionnaire

The Baby Care Questionnaire (BCQ) is a parent-report measure of parenting principles and practices during infancy. Parent-report measures offer many advantages, including access to difficult-to-observe situations, such as night-time sleeping practices, and cognitive processes, such as goals and principles (Miller, 2007). Parent-report methods are also cheaper and easier to administer, potentially increasing both sample size and utility. Parent-report measures have been used successfully to measure goals, expectations and beliefs about development (for example, Harwood, McLean, & Durkin, 2007; Keller et al., 2005; Miller-Loncar, Landry, Smith, & Swank, 2000). St James-Roberts (2007) identified a need for theoretically-driven studies to examine the measurable effects of various caregiving practices on infant outcomes. To our knowledge, the BCQ is the first comprehensive measure of principles and practices across caregiving domains.

The BCQ contains three sections: *Sleeping*, *Feeding* and *Soothing*. These three contexts of caregiving were chosen because in each of these domains parents are confronted with daily decisions, for example, when and where their baby should sleep, what and when to feed their baby, and when and how to respond to their infant's crying (Small, 1999). For each of these three caregiving contexts, the principles of structure and attunement are measured by parents’ endorsements of a series of statements, while practices are measured through checklists and quantitative questions (such as estimated duration). As noted in the previous section, structure and attunement are theoretically-driven constructs based on broader claims and empirical findings about how interactions between parents and their children shape development. The soothing section is the one section that asks about infant behaviour as well as parenting principles and practices. It seemed key to include reported crying duration due to a number of studies reporting that infant crying is related to caregiving (for example, Bell & Ainsworth, 1972; Hubbard & van Ijzendoorn, 1991; St James-Roberts et al., 2006). To ensure the BCQ can be used in longitudinal studies beginning during pregnancy and for comparisons between parents of healthy infants and infants “at risk”, the BCQ was designed to be valid for current and expectant parents, and for clinical and non-clinical samples.

To generate items for the statements assessing structure and attunement, reviews were conducted of the research literature on caregiving during infancy, parenting books and online sources of parenting advice, and discussions between parents on parenting websites and forums. After the initial generation of 58 statements, the theoretical constructs of structure and attunement were used to group items, and then adapt and select final items. Structure items were selected to measure parents’ endorsement of schedules and routines in infants’ daily lives and attunement items were selected to measure parents’ endorsement of infant cues and close physical contact in infants’ daily lives. Discussions resulted in the removal of 10 items due to the redundancy or complexity of items until consensus between the two authors was achieved on the 48 final items.

The suitability and readability of questions and items was then assessed using cognitive interviews. Cognitive interviews are used to examine potential breakdowns in how respondents understand, mentally process, and respond to questions in a questionnaire (Willis, 2005). Cognitive interviews are therefore useful in identifying questions, specific words or concepts that respondents have difficulty understanding, as well as questions that different respondents have different understandings of. We used a combination of think-aloud and verbal probing procedures (Campanelli, 1997) while 5 parents completed the BCQ. The interviewer asked the respondent to complete the BCQ while describing their thought processes but did not engage the respondent further unless the respondent stopped speaking. When such breaks occurred, the interviewer used a neutral probe (‘what does that make you think?) to remind the respondent to continue thinking aloud. On completion of the BCQ, the interviewer asked the respondent which items measured attitudes towards a number of phrases from parenting books (controlled crying, self-soothing and baby-led feeding). This verbal comprehension probe was used due to concerns about participant's understanding of such terminology.

Participants’ thoughts in response to items designed to measure structure and attunement highlighted problematic items and allowed modifications to enhance the clarity of the BCQ. Three difficulties occurred. First, participants had difficulty understanding the overall item (occurred in 4 items). For example, participants had difficulty understanding whether the item ‘Babies adjust to what they are fed’ reflected the amount or type of food and so was changed to ‘Babies adjust to the amount of milk/food available’. Second, participants had difficulty with specific words or concepts in the item (occurred in 4 items). For example, participants did not understand the use of the word *forced* in the item ‘Babies cannot be *forced* to sleep if they don’t want to’ and so was changed to ‘Babies know when they need to sleep’. Finally, different participants had different understandings of the item (occurred in 1 item). For example, ‘Strict sleeping routines deprive parents of spontaneity’ had different understandings – some focused on the effects routines had on their social life and others on the effect routines had on spontaneous interactions with their child. Therefore, two items were created: ‘Babies adjust when a parent's spontaneous night out keeps them up late’ and ‘Strict sleeping routines prevent parent(s) from enjoying their child’. Responses to the comprehension probe demonstrated that all participants understood the terminology from parenting advice books, regardless of whether they had read any of these books. Additionally, when items were reviewed, participants accurately identified statements referring to these behaviours and strategies.

Predictions based on the theoretical framework and past findings allowed examination of the psychometric properties of the BCQ subscales. We first hypothesised a two-factor model for the BCQ with the two components representing the parenting principles of structure and attunement. These BCQ subscales should show consistency within subscales at one time (internal consistency) and across time (test–retest reliability).

Individual differences in parenting principles were examined for structure and attunement by infant gender and parents’ status (current vs. expectant parents). Concurrent validity was tested by examining relations between principles and practices as well as principles and attributions of control over caregiving failures measured by the Parent Attribution Test (PAT; Bugental, Johnston, New, & Silvester, 1998). We hypothesised that principles would be related to practices. Specifically, we hypothesised that attunement would be related to more bed-sharing, breastfeeding and longer durations of reported holding; structure would be related to less bed-sharing, breastfeeding and shorter durations of reported holding. The interactive effect of structure and attunement was also examined for all parenting practices and infant crying. For parenting attributions, we hypothesised that principles would be associated with parents’ perceptions of caregiving failures. Specifically, we hypothesised that mothers with high perceived control scores – assigning more responsibility to the adult than child – would endorse attunement more and structure less. Although we report data from an initial (version 1) and a refined (version 2) form of the BCQ, all scores for structure and attunement are based on the items making up version 2.

## 2 Method

### 2.1 Participants and procedures

Participants were recruited from a UK database of families interested in participating in developmental psychology research, and by emails through mailing lists and online postings on UK parenting websites. Individuals were eligible if they were expecting a baby or had at least one child under 24 months old. Participants were asked to follow a link that led to an overview of the questionnaire(s) and were informed that pressing “next” was deemed as consent to participate. On pressing next, parents were asked if they were expectant or current parents. Parents that selected “my baby is not yet born” were directed to a version of the BCQ that only included items measuring parenting principles. All other parents went to a version of the BCQ that included parenting principles items and questions about parenting practices. Those participants who wanted to participate but did not have Internet access completed a paper version of the questionnaire(s) and returned questionnaires via freepost (*N* = 22).

Six hundred and forty-seven parents started completing the questionnaire(s) between April 2010 and April 2012. Participants with data missing from more than 30% of items were eliminated from subsequent analyses, resulting in a final sample of 610. There was no association between participants’ completion of the BCQ and whether their baby was born, *χ*^2^(1, *N* = 645) = 0.57, *p* = .494. For current parents, there were no differences between the infants of participants who completed and did not complete the questionnaire in terms of their age, *t*(529) = 0.86, *p* = .390, or gender, *χ*^2^(1, *N* = 535) = 3.57, *p* = .079. Additionally, there was no association between parents’ gender and completion of the BCQ, *χ*^2^(1, *N* = 223) = 3.08, *p* = .200.

Sample 1 (*N* = 346) only completed version 1 of the BCQ. Sample 2 (*N* = 216) completed version 2 of the BCQ. The test–retest sample (*N* = 48) completed version 2 of the BCQ at two time points separated by 4 to 6 weeks. This sample also completed the Parent Attribution Test at both time points. Infant and caregiver characteristics are summarised in Table 1 for the three samples. All procedures were approved by the School of Psychology Ethics Committee at Cardiff University.

### 2.2 Measures

#### 2.2.1 The Baby Care Questionnaire (BCQ) – version 1

Parenting principles are measured by respondents’ ratings of 48 items, consisting of two 24-item scales representing structure and attunement. Each section contains eight items designed to measure structure and eight items designed to measure attunement. Half of these items are designed to endorse the specific parenting principles and half to oppose. Items are rated on a 4-point Likert-type scale ranging from strongly disagree (1) to strongly agree (4). Parents are not given an option to select *not applicable* to prevent parents using this option despite having an opinion. Items can be left if parents truly did not have an opinion.

Parenting practices are measured by quantitative responses to three further items based on St James-Roberts et al.’s (2006) measure. In the sleeping section, parents are asked to indicate the number of nights, in the past seven, their infant slept in each location option. Sleeping options are a cot, a parent's bed, other, or a combination of locations. In the feeding section, parents are asked to indicate what they are feeding their infant from a list of breast milk, formula, expressed breast milk, milk-bank (during hospital stay) and solid food. Parents can indicate as many items as are relevant. The soothing section contains a quantitative question about infant behaviour. Parents are asked to report the estimated duration their infant cried for each day in the previous seven.

#### 2.2.2 The Baby Care Questionnaire (BCQ) – version 2

The second version of the BCQ contains the same items designed to measure structure and attunement. However, 18 items were dropped due to general concerns about singularity and specifically due to restricted responses from participants, low loadings (<.30), high complexity (multiple loadings) and intercorrelations between items (Field, 2005; Tabachnick & Fidell, 2007). All the final items of the BCQ are included in Table 2.

Version 2 includes the same items as version 1 to measure parents’ sleeping and feeding practices, and infant crying. However, parents are asked to report only on the previous three days for sleep location and infant cry duration. These items were reduced to the previous three days due to concerns raised by respondents about their accuracy across the past week on these items. In the feeding section, parents are also asked to report estimated duration of feeding for each of the previous three days. In the soothing section, parents are also asked to report estimated duration of holding for each of the previous three days. This holding item is not specific to the context of soothing but asks parents about times they were holding their infant, including times the infant was in a sling. Examples of parenting practice items are included in Appendix A.

#### 2.2.3 The Parent Attribution Test (PAT; Bugental et al., 1998)

The PAT assesses parents’ attributions about the relative influence of the parent versus the child on caregiving outcomes. Parents are presented with two hypothetical scenarios – they were looking after the neighbour's child for an afternoon and it either did or did not go well. As attributions for success have not been found to predict child or family outcomes (Bugental, 2011), we only focused on attributions for failure. Participants are asked to rate a series of possible reasons for caregiving failures on a 7-point rating scale ranging from 1 (not at all important) to 7 (very important). Items include ways the adult (*whether or not the adult liked children* and *the approach used*) or child (*the extent to which the child was stubborn* and *how little effort the child made)* have control over the outcome.

Attributional categories were calculated for factors that were: controllable by adults; uncontrollable by adults; controllable by child; uncontrollable by child. Participants who assigned high importance to self-controllable and low importance to self-uncontrollable were viewed as attributing high control to self over failure (ACF). The ACF subscale was an average of adult-controllable items and reversely scored adult-uncontrollable items, resulting in a score that could range from 1 to 7. Participants who assigned high importance to child-controllable and low importance to child-uncontrollable were viewed as attributing high control to children over failure (CCF). CCF was scored in the same way as ACF. Bugental (2011) recommended using a continuous perceived control over failure (PCF) variable rather than the traditional categorisation into low and high PCF. This continuous PCF variable is calculated by subtracting CCF from ACF. Higher scores reflect respondents attributing more responsibility to the adult rather than child in caregiving failures.

## 3 Results

### 3.1 Analysis plan

Prior to data analyses, variables were examined for the presence of outliers and normal distributions. Outliers were defined as 3.29 SD above or below the mean (Field, 2005). Any outliers identified were substituted with the value that reflected 3 SD above or below the mean. For sample 2 (completing version 2), the non-normality of duration of feeding and crying were resolved using a natural log transformation and the non-normality of duration of holding was resolved using a square root transformation. However, the non-normality of infant age was not resolved using transformations and therefore non-parametric tests were used for this variable. For ease of interpretation, all Tables and Figures depicting descriptive data use raw, rather than transformed, data.

The results are presented in four sections. First, we present a Principle Components Analysis (PCA; sample 1) and a Confirmatory Factor Analysis (CFA; sample 2) documenting the factor structure of the BCQ. Both the final PCA and the CFA were run on items that make up version 2 of the BCQ. Second, we present data about the reliability of the BCQ by reporting internal consistency coefficients (sample 1 and sample 2) and test–retest reliability (test–retest sample). This section also reports interrelations between the subscales (overall sample). Third, we present descriptive statistics about the subscales of the BCQ based on the overall sample (sample 1, sample 2 and T1 data for the test–retest sample). The final section presents two sets of analyses focusing on the validity of the BCQ. We explored relations between principles, practices and infant crying for the current parents in sample 2, as we had information about sleeping and feeding practices as well as feeding and holding durations for this sample. As expectant parents do not start using parenting practices until the birth of their child, they did not complete parenting practices items and were not included in analyses focusing on relations between principles and practices. In the second set of analyses focussing on validity, relations between principles in the BCQ and parents’ perceived control over caregiving failure in the PAT were examined in the test–retest sample.

### 3.2 Factor structure

To examine the factor structure of the BCQ, a PCA and CFA were run. A PCA with varimax rotation was conducted on the items designed to measure structure and attunement using SPSS version 16 (Norusis, 2008). Based on the results of the initial PCA, 18 items were dropped due to general concerns about singularity and specifically due to restricted responses from participants, low loadings, high complexity and intercorrelations between items (as noted in Section 2). Therefore, 30 items remained that were designed to measure structure and attunement. All analyses reported here – factor structure, descriptive statistics, reliability and validity – are based on these 30 items regardless of whether respondents completed version 1 or 2 of the BCQ.

**Fig. 1:**
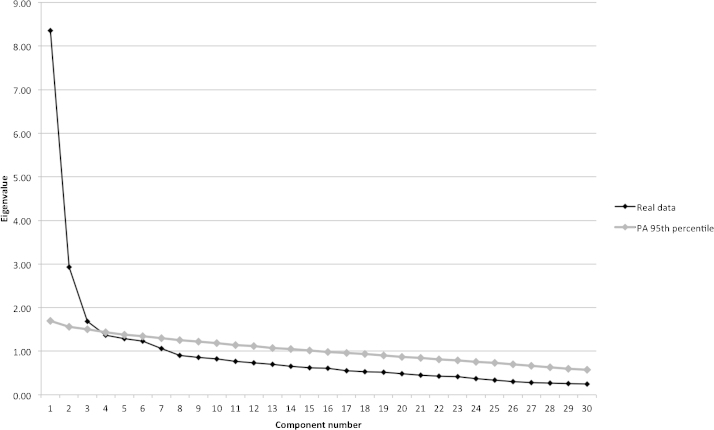
Scree plot depicting Eigenvalue against component number for real and random (PA) data.

The PCA was re-run with the remaining 30 items (that make up version 2 of the BCQ). The Kaiser-Meyer-Olkin (KMO) measure verified the sampling adequacy for the analysis; the overall KMO = .89 and KMO values for all individual items were above the acceptable level of .50. Bartlett's test of sphericity, *χ*^2^ (435) = 4001.34, *p* > .001, indicated that correlations were sufficiently large for PCA. An initial analysis was run to obtain eigenvalues for each component in the data. Seven components had eigenvalues over Kaiser's criterion of 1 and in combination explained 60% of the variance. However, this method is often criticised for retaining too many factors (Hayton, Allen, & Scarpello, 2004; O’Connor, 2000), so we used Horn's (1965) parallel analysis (PA) and Cattell's (1966) scree method to determine the number of components. Fig. 1 shows the scree plot of component number by eigenvalue for the real data and for random data with the same number of variables and sample size or PA 95th percentile data. This plot shows a clear inflexion at component 3 that justifies retaining component 2. Additionally, around component 3 the eigenvalues for the real data and random data have very similar values. Therefore two factors were retained in the final analysis. The PCA with varimax rotation was re-run specifying a two-factor solution. In combination, these two factors accounted for 37% of the variance. Table 2 shows the factor loadings after rotation. The items that cluster onto component 1, accounting for 21% of variance, represented endorsement of structure and component 2, accounting for 16% of variance, represented endorsement of attunement.

A CFA was conducted to confirm the two-factor structure of the BCQ using AMOS 18 (Arbuckle, 2010). The confirmatory model was set up so that items were free on their specific factor but restricted all other weights to 1. We used the full information maximum likelihood (FIML) approach to deal with the 3% of items with missing data, to avoid reduction in power or introducing bias through listwise deletion (Arbuckle, 2010; Enders, 2010). Correlations were added to account for shared variance of the shared context. Within the three contexts of the BCQ – sleeping, feeding and soothing – correlations were added between the errors for all items measuring each factor. For example, the errors for all items measuring structure in the sleeping section were correlated. The two-factor model generally demonstrated adequate fit, *χ*^2^(341) = 616.24, *p* < .001, *χ*^2^/df ratio = 1.81, root mean square error of approximation (RMSEA) = .06, Comparative fit index (CFI) = .87, Incremental fit index (IFI) = .88. A model was thought to show good fit if the *χ*^2^ test was not significant (*p* > .05), the CFI and IFI were .90 or above (Bentler, 1990; Marsh, Balla, & Hau, 1996) and the RMSEA was .06 or smaller (Hu & Bentler, 1999). Given the *χ*^2^ value is sensitive to small samples (Cheung & Rensvold, 2002) and the size of the correlations in the model (Miles & Shevlin, 2007), we gave greater weight to the incremental fit indices than to *χ*^2^. Factor loadings of items in the model are shown in Table 2.

### 3.3 Reliability and interrelations of the BCQ

Table 3 shows the internal consistency (*α*), test–retest reliability and intercorrelations of the structure and attunement subscales. The *α* coefficients for both subscales were above the .70 acceptable levels (Anastasi & Urbina, 1997; Kline, 2000). All items making up the structure and attunement subscales appeared worthy of retention. Test–retest reliability coefficients were calculated over a 4- to 6-week period for a sample of 48 mothers. These coefficients were in the acceptable range for both subscales, greater than *r*(48) = .70. There was a significant negative correlation between structure and attunement, accounting for 17% of the variance (see Table 3).

### 3.4 Descriptive statistics

Table 4 shows the means and standard deviations for the subscales of the BCQ for the overall sample as well as male and female infants, and current and expectant parents. Inferential statistics did not find any significant differences in endorsement of parenting principles by infant gender or parental status. Infants’ age was not related to scores on structure or attunement for current parents, *r*_*s*_(564) = .02, *p* = .717, *r*_*s*_(564) = −.01, *p* = .911, respectively.

### 3.5 Validity I: Effect of parenting principles on parenting practices and infant crying

Only current parents were used to examine the effect of parenting principles on parenting practices. Regression analyses were run with parenting practice as the criterion variable and centred-structure and centred-attunement (model 1), and structure × attunement interaction (model 2) as predictor variables. Table 5 presents the unstandardised regression coefficients (*B*), the standard error of the mean (*SE B*), and the standardised regression coefficients (*β*) for the predictor variables for each parenting practice. These analyses were run to see whether structure and attunement independently predicted parenting practices or if it was the interaction between structure and attunement that was important.

**Night-time sleeping practices.** Respondents indicated where their infants slept for each night in the three days preceding questionnaire completion. Bed-sharing items were: slept in a parent's bed all night; moved from parent's bed to a cot; moved from a cot to a parent's bed; and moved from somewhere other than a parent's bed or cot to a parent's bed. Bed-sharing items for each respondent were summed to create an overall number of nights bed-sharing variable that could range from 0 to 3. Twenty-eight per cent of parents reported at least 1 night of bed-sharing and the mean number of nights was 0.60 nights (*SD* = 1.07).

Model 1 – structure and attunement – was significant, *R*^*2*^ = .05, *F*(2, 203) = 4.92, *p* = .008, and was not improved by adding the structure × attunement interaction variable, Δ*R*^*2*^ = .00, *F*(1, 202) = 0.13, *p* = .721. Attunement was the only independent predictor of number of nights of bed-sharing – higher attunement predicted more nights of bed-sharing, *β* = .19, *t*(204) = −2.14, *p* = .033.

**Feeding practices.** Respondents feeding their infant breast milk (either exclusively or in combination with formula or solids) were assigned a 1 and respondents not feeding their infant breast milk were assigned 0 for the breastfeeding category. Forty-one per cent of respondents were feeding their infant breast milk. A categorical regression was run with structure, attunement and structure × attunement interaction variables as predictors and breastfeeding category (1 = yes, 0 = no) as the outcome variable. A significant model was found, *R*^*2*^ = .15, *F*(18, 187) = 1.89, *p* = .019. Attunement was the only independent predictor of breastfeeding – higher attunement predicted breastfeeding, *β* = .20, *F*(6, 187) = 2.36, *p* = .032. Structure approached significance as a predictor – lower structure was related, at trend levels, to breastfeeding, *β* = −.19, *F*(6, 187) = 2.11, *p* = .054.

**Reported duration of feeding and holding.** Average durations of feeding and holding were calculated in minutes based on the daily estimates for the preceding three days. For average duration of feeding, model 1 – structure and attunement – was not significant, *R*^*2*^ = .01, *F*(2, 203) = 0.66, *p* = .517, and was not improved by adding the structure × attunement interaction variable, Δ*R*^*2*^ = .01, *F*(1, 202) = 1.70, *p* = .194. For average duration of holding, model 1 – structure and attunement – was significant, *R*^*2*^ = .09, *F*(2, 197) = 9.39, *p* < .001, and was not improved by adding the structure × attunement interaction variable, Δ*R*^*2*^ = .00, *F*(1, 196) = 0.02, *p* = .904. Attunement was the only independent predictor of number of average duration of holding – higher attunement predicted longer durations of holding (*β* = .22, *t*(198) = 2.91, *p* = .004).

**Reported duration of infant crying.** Average duration of crying was calculated in minutes based on the daily estimates for the preceding three days. Model 1 – structure and attunement – was not significant, *R*^*2*^ = .02, *F*(2, 198) = 2.46, *p* = .088, and was improved by adding the structure × attunement interaction variable, Δ*R*^*2*^ = .02, *F*(1, 197) = 4.56, *p* = .034. Model 2 – including the interactive variable – was significant, *R*^*2*^ = .05, *F*(3, 197) = 3.19, *p* = .025. The interaction between structure and attunement was the only independent predictor of average duration of crying (*β* = .16, *t*(200) = 2.14, *p* = .034).

**Fig. 2:**
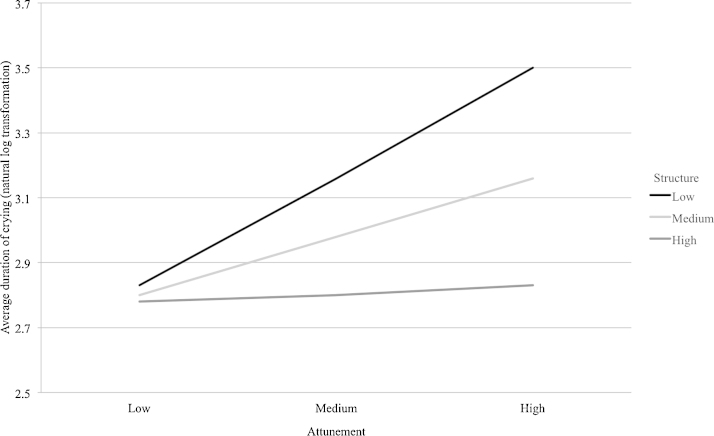
Interaction between structure and attunement in predicting average duration of crying (with natural log transformation).

Fig. 2 presents a graphical representation of the interaction between structure and attunement (created using the ModGraph-I programme; Jose, 2008). In order to further understand this significant interaction, simple slopes analyses were used to understand which slopes differed significantly from 0 (or from a flat line). The slopes for high levels of structure, *t*(197) = −0.18, *p* = .859, and medium levels of structure, *t*(197) = −1.30, *p* = .196, did not differ from 0. However, the slope for low levels of structure differed significantly from 0, *t*(197) = −2.41, *p* = .017. At low levels of structure, average duration of crying increased as attunement level increased. Therefore, structure appears to buffer the effect of attunement on average duration of crying.

### 3.6 Validity II: Perceived control over caregiving failure and parenting principles

As parents who placed more responsibility on the child than the adult for caregiving failures were more directive in their behaviour (Bugental & Johnston, 2000; Guzell & Vernon-Feagans, 2004), we predicted perceived control over caregiving failures should be related to structure and attunement. Associations between parenting principles and perceived control over caregiving failures, measured by the PAT, were therefore examined in attempts to confirm the validity of the BCQ. Both adult control over caregiving failures (ACF), child control over caregiving failures (CCF) and perceived control over failures (PCF) showed test–retest reliability, *r*(48) = .35, *p* = .014, *r*(48) = .58 *p* < .001, *r*(48) = .31, *p* = .031, respectively. Scores on structure, attunement, CCF, ACF and PCF were averaged across the two time points. Table 6 presents means and standard deviations of average CCF, ACF and PCF, as well as the correlations between these scales and the parenting principles structure and attunement. PCF was negatively related to structure and positively related to attunement – respondents who placed more responsibility on the child in caregiving failures endorsed structure more and attunement less. When examining the two underlying dimensions of PCF – ACF and CCF – attributions about the child appeared to be related to parenting principles, with structure positively related and attunement negatively related to CCF. Adult attributions were not related to structure or attunement.

## 4 Discussion

The BCQ is the first comprehensive measure of parenting principles and practices across caregiving domains during infancy. We hypothesised that parents of infants are guided by two principles, structure and attunement, and that these principles are related to specific practices such as feeding, holding and night-time sleeping. We investigated the BCQ's psychometric properties, reliability, and validity. The BCQ's two-factor structure was confirmed by a principal components analysis and a confirmatory factor analysis. These two subscales showed good internal consistency and test–retest reliability, showing consistency across items of subscales when completed at a single time and for the overall subscales when completed across time.

We examined the validity of the BCQ in three steps. First, early piloting, using cognitive interviews, demonstrated the BCQ's content validity by showing that most items were read, processed and answered in the intended way. Minor adjustments were made to problematic items to enhance clarity. Second, we examined relations between principles and practices in a larger sample. Attunement was positively related to bed-sharing, breastfeeding and holding. Structure was negatively related to breastfeeding at trend levels, and structure appeared to buffer the effects of attunement on reported duration of crying. That is, at low levels of structure, average duration of reported crying increased as attunement level increased. Observed relations between parenting principles and practices provide further support for the validity of the BCQ given principles must relate to practices to have an effect on children (Holden & Edwards, 1989). Third, based on previous studies, we compared parenting principles during infancy with parents’ perceptions of caregiving failures. Respondents who placed more responsibility on the adult than the child for caregiving failures endorsed attunement more and structure less. These findings appeared to be driven by respondents’ attributions about the child – related positively with structure and negatively with attunement – rather than attributions about the adult, which were not related to structure or attunement.

Hypothesised relations between structure and bed-sharing were not observed. Bed-sharing was only reported by 28% of respondents. This low rate of bed-sharing may be the reason for the lack of associations between bed-sharing and structure. Bed-sharing, among other practices, is an example of a parenting practice that is not always possible given situational and cultural constraints. In the UK, parents are advised to place their infants to sleep in a cot near their parents’ bed, not to fall asleep with their baby on a sofa or armchair and not to share a bed if they had been drinking, smoking or taking drugs (NHS choices, 2012; UNICEF UK Baby Friendly Initiative, 2013). The lack of relations between sleeping practices and structure may therefore be a reflection of the restrictions of the cultural contexts. In addition, bed-sharing comprises a variety of behaviours, with different parents having different motivations for this parenting practice (Ball & Volpe, 2013; Volpe et al., 2013). Therefore, bed-sharing cannot be considered in absence of the wider cultural beliefs of the mother. Accordingly, relations between bed-sharing and parenting principles should be compared in cultures where bed-sharing is necessary and/or expected and in cultures where bed-sharing is neither restricted nor expected.

### 4.1 Implications

The BCQ provides an important new framework and measure to explore the role of parenting principles and practices during infancy. Unlike other measures, the BCQ measures how parents meet their infant's needs across caregiving domains and therefore provides a comprehensive measure of practices and principles. As assessment tools are required for real progress to be made in testing and refining theory (Davies, Forman, Rasi, & Stevens, 2002), we believe this measure is needed in order to start addressing a broad range of significant and neglected questions during infancy.

The BCQ will be a valuable tool, not only in characterising early parenting principles and practices, but also in investigating the influence of infant characteristics, environmental factors and adult cognitions. One environmental factor is culture. This report only collects data from a UK sample; however, previous work suggests that cultures should differ in the relative importance they ascribe to structure and attunement during infancy (for example, Gantley, Davies, & Murcott, 1993; Hewlett, Lamb, Shannon, Leyendecker, & Scholmerich, 1998; St James-Roberts et al., 2006). Practices also differ by culture – for example, bed-sharing is common in non-industrialized societies (Blair, 2010; Mindell et al., 2010). Thus, once established as a valid tool in other cultures, the BCQ can be used to document cross-cultural variations in principles and improve our understanding of how principles and practices are related within certain cultural contexts.

### 4.2 Limitations and future directions

The cross-sectional data in this report cannot demonstrate how consistent these parenting principles and practices are across time. A longitudinal design would also allow a clearer understanding of change over time. Parenting principles did not vary with infant age; however, individual parents’ endorsement of different principles may still change over time or in response to infants’ behaviours, such as crying. Further research using the BCQ in a longitudinal design would allow a better understanding of changes in principles based on experience with infants as well as infants’ age and behaviour, and parity of parents.

An additional benefit of a longitudinal design would be to understand differences in parenting principles before or at the onset of parenting and then once parents are established in their caregiving role. The BCQ was designed for current and expectant parents. These two groups did not differ on their endorsement of structure or attunement. The current cross-sectional design does not provide answers to questions about whether principles change once parenting begins, or if principles developed during pregnancy persist throughout infancy. As previous studies have used such decisions to group parents during pregnancy (see St James-Roberts et al., 2006), future work should examine the reliability and validity of expectant parents reporting their principles.

A second limitation is the reliance on parent-report measures. The BCQ was designed as a parent-report measure, as parenting principles and practices are difficult to observe due to their cognitive focus and personal nature. However, a key step in verifying validity is examining the belief-behaviour match. This process would allow us to confirm the BCQ measures the principles and practices reported. Some researchers claim exploring these relations is the most important validation process (for example, Dekovic, Janssens, & Gerris, 1991; Miller, 2007).

The final limitation of this study was the sample's diversity. The current report provides initial validation of the BCQ in the UK. UK parents vary in the extent to which they endorse structure and attunement, and in their choices of parenting practices. However, as this study required Internet access – and so not all parents could participate – the BCQ needs further validation in a more diverse UK sample. Unfortunately, we did not collect data on the ethnicity, socioeconomic status or parity of parents. Therefore, an important next step would be to understand the role of these variables on these parenting principles and practices. In addition, the BCQ needs to be validated in a wider number of cultures.

The BCQ, and data collected from such a measure, will be relevant to practitioners interested in promoting healthy practices, such as Kangaroo care for preterm infants or breastfeeding. Future studies should include risk samples at differing stages of development in order, for example, to better our understanding of the early influence of the NICU stay on parenting principles and practices. Alternative risk groups include parents of siblings of children with Autism Spectrum Disorder (ASD) or mothers with postnatal depression.

## 5 Conclusions

The BCQ provides a new measure to explore the role of parenting principles and practices during infancy. By providing a measure suitable for infancy, it will allow investigation of the long-term effects of these early principles and practices on child social and cognitive outcomes. Despite fierce debate on the benefits and costs of different caregiving approaches, there is little empirical evidence documenting the long-term outcomes for children (St James-Roberts, 2007). Once the implications of early caregiving principles are better understood, practitioners will be able to give more informed advice to parents on the benefits and costs of different caregiving approaches, which can be tailored to the needs of the parents and their infants.

## Figures and Tables

**Figure:**
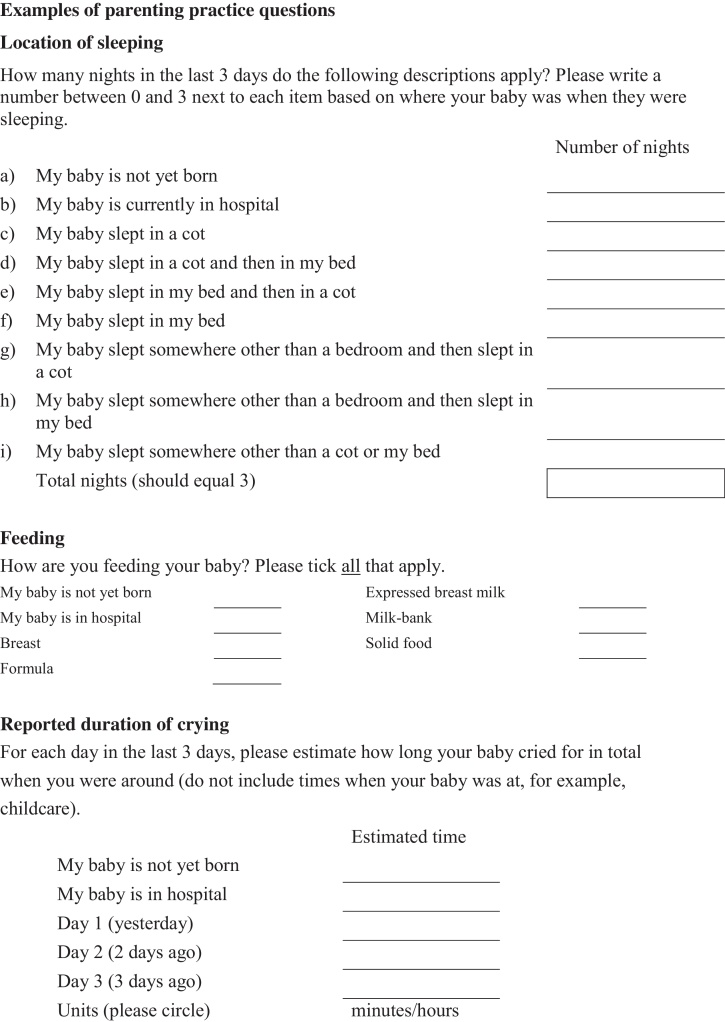


**Table 1:** Caregiver and infant characteristics of respondents in sample 1, sample 2, and test–retest sample.

	Sample 1	Sample 2	Test–retest sample
Caregiver gender			
Female (%)	N/A	98	100
Male (%)	N/A	2	0
Caregiver status			
Expectant (%)	14	4	0
Current (%)	86	96	100
Infant gender			
Female (%)	47	47	48
Male (%)	53	53	52
Infant age (months)	0.00–23.00 Mean = 11.31 (SEM = 0.36)	0.00–23.00 Mean = 9.43 (SEM = 0.36)	1.00–19.00 Mean = 8.71 (SEM = 0.73)

*Note*. *N*s for sample 1, sample 2 and test–retest sample were 344, 216 and 48, respectively. These *N*s are excluding the 37 participants with data missing from more than 30% of items so were treated as missing data.

**Table 2:** Factor loadings of the Principal Components Analysis (PCA) and Confirmatory Factor Analysis (CFA) of the Baby Care Questionnaire.

	Statement	PCA (CFA)	
		Structure	Attunement
Sleeping			
1.	Babies can have a good night's sleep regardless of scheduling	−.47 (−.45)	.19
2.	Strict sleeping routines prevent parent(s) from enjoying their child.	−.68 (−.65)	.18
3.	Sleeping schedules make babies unhappy	−.62 (−.59)	.09
4.	It is important to introduce a sleeping schedule as early as possible	.63 (.72)	−.29
5.	Babies benefit from a quiet room to sleep	.34 (.39)	.11
6.	Babies benefit from a fixed napping/sleeping schedule	.66 (.73)	−.16
7.	Some days, babies need more or less sleep than other days	−.01	.32 (.34)
8.	Babies benefit from physical contact with parent(s) when they wake during the night	−.29	.58 (.60)
9.	When babies cry in the night to check if someone is near, it is best to leave them	.08	−.71 (−.65)
Eating			
1.	Implementing feeding/eating schedules leads to a calm and content baby	.64 (.66)	−.24
2.	Feeding/eating routines are difficult (easy) to follow	−.56 (.48)	.13
3.	One danger of feeding/eating schedules is that babies might not get enough to eat	−.57 (.55)	.21
6.	Following feeding/eating routines prevents parent(s) from enjoying parenthood to the full	−.63 (−.70)	.05
7.	It is important to introduce a feeding/eating schedule as early as possible	.51 (.66)	−.38
10.	Babies will not follow feeding/eating schedules	−.67 (−.53)	−.06
4.	Parent(s) should find a pattern of feeding/eating that suits the baby	−.05	.48 (.38)
5.	Baby-led feeding leads to behavioural and sleep problems	.28	−.52 (−.54)
8.	Offering milk/food to a baby is a good way to test whether she/he is hungry	−.12	.38 (.28)
9.	Babies will eat whenever milk/food is offered even if they are not hungry	−.08	−.46 (−.17)
Crying			
1.	Babies with regular schedules spend less time crying	.66 (.66)	−.25
2.	Babies cry no matter what their routines	−.35 (−.25)	.20
4.	Routines lead to more crying	−.71 (−.70)	.01
9.	Having a set routine helps an upset baby calm down	.65 (.62)	−.20
10.	Babies with regular schedules cry just as much as babies without regular schedules	−.53 (−.51)	.16
3.	Parent(s) should delay responding to a crying baby	.17	−.77 (−.58)
5.	It is a good idea to have a set time you leave a baby to calm herself/himself down, and increase this amount of time each week	.30	−.61 (−.57)
6.	Physical contact such as stroking or rocking helps a baby to be calm	−.05	.60 (.43)
7.	Holding babies frequently during the day makes them more demanding	.20	−.57 (−.46)
8.	Responding quickly to a crying baby leads to less crying in the long run	−.18	.66 (.64)
11.	Leaving a baby to cry can cause emotional insecurity	−.21	.65 (.55)

*Note*. *N*s for sample 1 (PCA) and sample 2 (CFA) were 344 and 216, respectively. These *N*s are excluding the 37 participants with data missing from more than 30% of items. Factor loadings > 29 are in boldface. Factor loadings without parentheses are from the PCA and within parentheses are from the CFA. Item 2 of the eating section read *… are difficult to follow* in version 1 and *… are easy to follow* in version 2.

**Table 3:** Intercorrelations, internal consistency and test–retest reliability of the subscales of the Baby Care Questionnaire.

Subscale	Intercorrelations	Internal consistency		Test–retest
	2. Attunement	Sample 1	Sample 2	*r*_*s*_(48)
1. Structure	−.47***	.89	.91	.91***
2. Attunement	–	.83	.81	.83***

*Note*. *N*s for the factor intercorrelation, sample 1, sample 2, and test–retest sample were 608, 344, 216 and 48, respectively. These *N*s are excluding the 37 participants with data missing from more than 30% of items so were treated as missing data. *p < .05. **p < .01.

*** *p* < .001.

**Table 4:** Parenting principles in the Baby Care Questionnaire means and standard deviations for the overall sample, by each gender and by parent's status.

	Overall	Infant gender differences		Parent status differences	
		Boys	Girls	Current	Expectant
	*M* (*SD*)	*M* (*SD*)	*M* (*SD*)	*M* (*SD*)	*M* (*SD*)
Structure	2.74 (0.50)	2.74 (0.53)	2.73 (0.51)	2.74 (0.47)	2.76 (0.38)
Attunement	2.98 (0.50)	2.97 (0.35)	3.00 (0.51)	2.99 (0.47)	2.90 (0.30)

*Note*. *N*s for the overall sample, boys, girls, current and expectant were 624, 306, 292, 564, 58, respectively. These *N*s are excluding the 37 participants with data missing from more than 30% of items so were treated as missing data.

**Table 5:** Predictors of parenting practices.

	Structure		Attunement		Structure × attunement		Δ*R*^*2*^
	*B (SE)*	*β*	*B (SE)*	*β*	*B (SE)*	*β*	
Nights bed-sharing							
Model 1	−0.14 (0.18)	−.06	0.54 (0.22)	.19*			.05**
Model 2	−0.15 (0.19)	−.06	0.54 (0.22)	.19*	0.14 (0.39)	.03	.00
Feeding category		−.19^a^		.20*		−.20	.15*
Feeding duration							
Model 1	0.16 (0.14)	.09	0.09 (0.17)	.04			.01
Model 2	0.12 (0.15)	.07	0.11 (0.17)	.05	0.40 (0.31)	.10	.01
Holding duration							
Model 1	−1.28 (0.78)	−.12	2.70 (0.93)	.22**			.09***
Model 2	−1.26 (0.81)	−.12	2.69 (0.94)	.22**	−0.20 (1.66)	−.01	.00
Crying duration							
Model 1	−0.30 (0.21)	−.11	−0.53 (0.25)	−.17*			.02
Model 2	−0.41 (0.21)	−.15	−0.48 (0.25)	−.15	0.93 (0.44)	.16*	.02*

a *p* = .054.

* *p* < .05.

** *p* < .01.

*** *p* < .001.

**Table 6:** Means and standard deviations for the subscales of the Parent Attribution Test and their associations with parenting principles measured by the Baby Care Questionnaire.

	*Mean* (*SD*)	Structure (*r*)	Attunement (*r*)
Adult control over failure (ACF)	4.28 (0.49)	.02	.16
Child control over failure (CCF)	3.55 (0.52)	.36*	−.35*
Perceived control over failure (PCF)	0.78 (0.60)	−.36*	.47**

*Note*. PCF = ACF–CCF.

* *p* < .05.

** *p* < .001.
